# *N*-(3,4-Dimethyl­phen­yl)-2,4-dimethyl­benzene­sulfonamide

**DOI:** 10.1107/S1600536810014418

**Published:** 2010-04-24

**Authors:** B. Thimme Gowda, Sabine Foro, P. G. Nirmala, Hartmut Fuess

**Affiliations:** a Department of Chemistry, Mangalore University, Mangalagangotri 574 199, Mangalore, India; b Institute of Materials Science, Darmstadt University of Technology, Petersenstrasse 23, D-64287 Darmstadt, Germany

## Abstract

In the title compound, C_16_H_19_NO_2_S, the dihedral angle between the aromatic rings is 47.2 (2)°. The crystal structure features zigzag *C*(4) chains linked by N—H⋯O hydrogen bonds.

## Related literature

For the preparation of the title compound, see: Savitha & Gowda (2006 ▶). For our study on the effect of substituents on the structures of *N*-(ar­yl)aryl­sulfonamides, see: Gowda *et al.* (2009*a* ▶,*b* ▶); Nirmala *et al.* (2010 ▶). For related structures, see: Gelbrich *et al.* (2007 ▶); Perlovich *et al.* (2006 ▶).
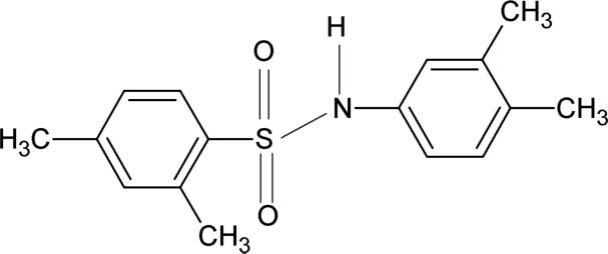


## Experimental

### 

#### Crystal data

C_16_H_19_NO_2_S
*M* *_r_* = 289.38
Monoclinic, 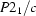

*a* = 9.732 (1) Å
*b* = 15.045 (2) Å
*c* = 10.425 (1) Å
β = 100.10 (1)°
*V* = 1502.8 (3) Å^3^
*Z* = 4
Cu *K*α radiation
μ = 1.92 mm^−1^
*T* = 299 K
0.45 × 0.38 × 0.28 mm

#### Data collection

Enraf–Nonius CAD-4 diffractometer
Absorption correction: ψ scan (North *et al.*, 1968 ▶) *T*_min_ = 0.479, *T*_max_ = 0.616
2858 measured reflections
2672 independent reflections
2265 reflections with *I* > 2σ(*I*)
*R*_int_ = 0.047
3 standard reflections every 120 min  intensity decay: 1.0%

#### Refinement

*R*[*F* ^2^ > 2σ(*F* ^2^)] = 0.070
*wR*(*F* ^2^) = 0.338
*S* = 1.59
2672 reflections
185 parameters
H-atom parameters constrained
Δρ_max_ = 0.71 e Å^−3^
Δρ_min_ = −0.71 e Å^−3^

### 

Data collection: *CAD-4-PC* (Enraf–Nonius, 1996 ▶); cell refinement: *CAD-4-PC*; data reduction: *REDU4* (Stoe & Cie, 1987 ▶); program(s) used to solve structure: *SHELXS97* (Sheldrick, 2008 ▶); program(s) used to refine structure: *SHELXL97* (Sheldrick, 2008 ▶); molecular graphics: *PLATON* (Spek, 2009 ▶); software used to prepare material for publication: *SHELXL97*.

## Supplementary Material

Crystal structure: contains datablocks I, global. DOI: 10.1107/S1600536810014418/bt5248sup1.cif

Structure factors: contains datablocks I. DOI: 10.1107/S1600536810014418/bt5248Isup2.hkl

Additional supplementary materials:  crystallographic information; 3D view; checkCIF report

## Figures and Tables

**Table 1:** Hydrogen-bond geometry (Å, °)

*D*—H⋯*A*	*D*—H	H⋯*A*	*D*⋯*A*	*D*—H⋯*A*
N1—H1*N*⋯O2^i^	0.86	2.41	2.976 (4)	124

Symmetry code: (i) 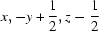.

**Fig. 1.**
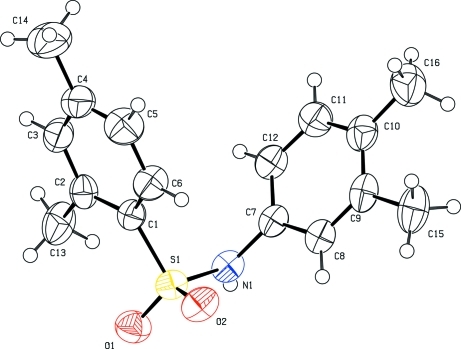
Molecular structure of (I), showing the atom labelling scheme and displacement ellipsoids are drawn at the 50% probability level. The H atoms are represented as small spheres of arbitrary radii.

**Fig. 2.**
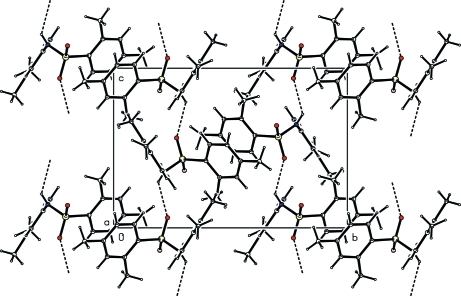
Molecular packing of (I) with hydrogen bonding shown as dashed lines.

## supplementary crystallographic information

### Comment

As part of a study of substituent effects on the structures of *N*-(aryl)arylsulfonamides (Gowda *et al.*, 2009*a*,*b*; Nirmala *et al.*, 2010),in the present work, the structure of 2,4-dimethyl-*N*-(3,4-dimethylphenyl)benzenesulfonamide (I) has been determined (Fig. 1). The conformation of the N—C bond in the C—SO_2_—NH—C segment of the structure has gauche torsions with respect to the S═O bonds. The molecule in (I) is bent at the *S*- atom with the C—SO_2_—NH—C torsion angle of 57.4 (3)°, compared to the values of 70.1 (2) and -66.0 (2)° in the two independent molecules of 2,4-dimethyl-*N*-(2,3-dimethylphenyl)benzenesulfonamide (II), 66.5 (2)° in 2,4-dimethyl-*N*-(2,4-dimethylphenyl)benzenesulfonamide (III) , 53.9 (2)° in 2,4-dimethyl-*N*-(3,5-dimethylphenyl)benzenesulfonamide (IV) and 46.1 (3)° (glide image of molecule 1) and 47.7 (3)° (molecule 2) in the two independent molecules of 2,4-dimethyl-*N*- (phenyl)benzenesulfonamide (V).

The sulfonyl and the anilino benzene rings in (I) are tilted relative to each other by 47.2 (2)°, compared to the values of 41.5 (1) and 43.8 (1)° in the two molecules of (II), 41.0 (1)° in (III), 82.1 (1)° in (IV) and 67.5 (1)° (molecule 1) and 72.9 (1)° (molecule 2) in (V),

The remaining bond parameters in (I) are similar to those observed in (II), (III), (IV), (V) and other aryl sulfonamides (Perlovich *et al.*, 2006; Gelbrich *et al.*, 2007). The crystal packing of molecules in (I) through N—H···O(S) hydrogen bonds (Table 1) is shown in Fig.2.

### Experimental

The solution of *m*-xylene (10 ml) in chloroform (40 ml) was treated dropwise with chlorosulfonic acid (25 ml) at 0 ° C. After the initial evolution of hydrogen chloride subsided, the reaction mixture was brought to room temperature and poured into crushed ice in a beaker. The chloroform layer was separated, washed with cold water and allowed to evaporate slowly. The residual 2,4-dimethylbenzenesulfonylchloride was treated with 3,4-dimethylaniline in the stoichiometric ratio and boiled for ten minutes. The reaction mixture was then cooled to room temperature and added to ice cold water (100 ml). The resultant solid 2,4-dimethyl-*N*- (3,4-dimethylphenyl)benzenesulfonamide was filtered under suction and washed thoroughly with cold water. It was then recrystallized to constant melting point from dilute ethanol. The purity of the compound was checked and characterized by recording its infrared and NMR spectra (Savitha & Gowda, 2006). Prism like yellow single crystals used in X-ray diffraction studies were grown in ethanolic solution by slow evaporation at room temperature.

### Refinement

The H atoms were positioned with idealized geometry using a riding model (C—H = 0.93–0.96 Å, N—H = 0.86 Å) and were refined with isotropic displacement parameters  set to 1.2 times of the *U*_eq_ of the parent atom. To improve the values of R1, wR2, and GOOF,   six reflections (-3 4 7, -3 9 3, 0 9 5, -8 4 3, 3 11 1, -8 4 1) were omitted from the refinement.

### Crystal data

**Table:**

C_16_H_19_NO_2_S	*F*(000) = 616
*M**_r_* = 289.38	*D*_x_ = 1.279 Mg m^−^^3^
Monoclinic, *P*2_1_/*c*	Cu *K*α radiation, λ = 1.54180 Å
Hall symbol: -P 2ybc	Cell parameters from 25 reflections
*a* = 9.732 (1) Å	θ = 4.6–22.4°
*b* = 15.045 (2) Å	µ = 1.92 mm^−^^1^
*c* = 10.425 (1) Å	*T* = 299 K
β = 100.10 (1)°	Prism, yellow
*V* = 1502.8 (3) Å^3^	0.45 × 0.38 × 0.28 mm
*Z* = 4	

### Data collection

**Table:**

Enraf–Nonius CAD-4 diffractometer	2265 reflections with *I* > 2σ(*I*)
Radiation source: fine-focus sealed tube	*R*_int_ = 0.047
graphite	θ_max_ = 67.0°, θ_min_ = 4.6°
ω/2θ scans	*h* = −11→11
Absorption correction: ψ scan (North *et al.*, 1968)	*k* = −17→0
*T*_min_ = 0.479, *T*_max_ = 0.616	*l* = −12→1
2858 measured reflections	3 standard reflections every 120 min
2672 independent reflections	intensity decay: 1.0%

### Refinement

**Table:**

Refinement on *F*^2^	Primary atom site location: structure-invariant direct methods
Least-squares matrix: full	Secondary atom site location: difference Fourier map
*R*[*F*^2^ > 2σ(*F*^2^)] = 0.070	Hydrogen site location: inferred from neighbouring sites
*wR*(*F*^2^) = 0.338	H-atom parameters constrained
*S* = 1.59	*w* = 1/[σ^2^(*F*_o_^2^) + (0.2*P*)^2^] where *P* = (*F*_o_^2^ + 2*F*_c_^2^)/3
2672 reflections	(Δ/σ)_max_ = 0.042
185 parameters	Δρ_max_ = 0.71 e Å^−^^3^
0 restraints	Δρ_min_ = −0.71 e Å^−^^3^

### Special details

**Table:**

Geometry. All esds (except the esd in the dihedral angle between two l.s. planes) are estimated using the full covariance matrix. The cell esds are taken into account individually in the estimation of esds in distances, angles and torsion angles; correlations between esds in cell parameters are only used when they are defined by crystal symmetry. An approximate (isotropic) treatment of cell esds is used for estimating esds involving l.s. planes.
Refinement. Refinement of *F*^2^ against ALL reflections. The weighted *R*-factor wR and goodness of fit *S* are based on *F*^2^, conventional *R*-factors *R* are based on *F*, with *F* set to zero for negative *F*^2^. The threshold expression of *F*^2^ > σ(*F*^2^) is used only for calculating *R*-factors(gt) etc. and is not relevant to the choice of reflections for refinement. *R*-factors based on *F*^2^ are statistically about twice as large as those based on *F*, and *R*- factors based on ALL data will be even larger.

### Fractional atomic coordinates and isotropic or equivalent isotropic displacement parameters (Å^2^)

**Table:**

	*x*	*y*	*z*	*U*_iso_*/*U*_eq_	
S1	−0.01170 (9)	0.29486 (6)	0.42947 (8)	0.0414 (5)	
O1	−0.1509 (3)	0.3027 (2)	0.3594 (3)	0.0581 (9)	
O2	0.0109 (3)	0.2694 (2)	0.5633 (3)	0.0539 (9)	
N1	0.0626 (3)	0.2187 (2)	0.3527 (3)	0.0420 (8)	
H1N	0.0143	0.1909	0.2880	0.050*	
C1	0.0835 (4)	0.3947 (2)	0.4209 (4)	0.0407 (9)	
C2	0.0551 (4)	0.4535 (3)	0.3159 (4)	0.0462 (10)	
C3	0.1420 (5)	0.5271 (3)	0.3193 (5)	0.0551 (11)	
H3	0.1251	0.5673	0.2507	0.066*	
C4	0.2520 (5)	0.5435 (3)	0.4192 (5)	0.0571 (12)	
C5	0.2745 (6)	0.4854 (3)	0.5202 (6)	0.0658 (13)	
H5	0.3478	0.4954	0.5889	0.079*	
C6	0.1904 (5)	0.4114 (3)	0.5229 (4)	0.0547 (11)	
H6	0.2062	0.3729	0.5938	0.066*	
C7	0.2084 (4)	0.1981 (2)	0.3928 (4)	0.0406 (9)	
C8	0.2517 (4)	0.1470 (3)	0.5026 (4)	0.0460 (10)	
H8	0.1860	0.1251	0.5494	0.055*	
C9	0.3919 (5)	0.1279 (3)	0.5439 (4)	0.0501 (10)	
C10	0.4903 (5)	0.1578 (3)	0.4705 (5)	0.0541 (11)	
C11	0.4431 (5)	0.2072 (3)	0.3588 (5)	0.0578 (12)	
H11	0.5072	0.2266	0.3084	0.069*	
C12	0.3043 (5)	0.2284 (3)	0.3204 (4)	0.0492 (10)	
H12	0.2757	0.2628	0.2464	0.059*	
C13	−0.0607 (6)	0.4423 (4)	0.2013 (5)	0.0685 (14)	
H13A	−0.1470	0.4619	0.2245	0.082*	
H13B	−0.0683	0.3808	0.1767	0.082*	
H13C	−0.0408	0.4770	0.1295	0.082*	
C14	0.3438 (7)	0.6239 (4)	0.4164 (9)	0.094 (2)	
H14A	0.2915	0.6702	0.3666	0.113*	
H14B	0.4227	0.6084	0.3772	0.113*	
H14C	0.3756	0.6444	0.5038	0.113*	
C15	0.4395 (6)	0.0747 (3)	0.6666 (5)	0.0719 (15)	
H15A	0.4804	0.0199	0.6449	0.086*	
H15B	0.3609	0.0622	0.7080	0.086*	
H15C	0.5075	0.1083	0.7250	0.086*	
C16	0.6417 (6)	0.1387 (4)	0.5107 (7)	0.0804 (18)	
H16A	0.6534	0.0807	0.5496	0.096*	
H16B	0.6828	0.1825	0.5729	0.096*	
H16C	0.6865	0.1406	0.4358	0.096*	

### Atomic displacement parameters (Å^2^)

**Table:**

	*U*^11^	*U*^22^	*U*^33^	*U*^12^	*U*^13^	*U*^23^
S1	0.0466 (7)	0.0476 (7)	0.0298 (7)	−0.0046 (3)	0.0060 (4)	0.0006 (3)
O1	0.0496 (19)	0.065 (2)	0.058 (2)	−0.0046 (13)	0.0062 (15)	0.0016 (14)
O2	0.075 (2)	0.0593 (18)	0.0301 (16)	−0.0065 (14)	0.0176 (14)	0.0018 (11)
N1	0.0517 (19)	0.0441 (17)	0.0270 (15)	−0.0076 (13)	−0.0018 (13)	−0.0048 (12)
C1	0.046 (2)	0.0384 (19)	0.0377 (19)	0.0013 (14)	0.0077 (15)	−0.0023 (14)
C2	0.052 (2)	0.043 (2)	0.043 (2)	0.0089 (16)	0.0090 (17)	−0.0022 (16)
C3	0.065 (3)	0.044 (2)	0.060 (3)	0.0053 (19)	0.020 (2)	0.0052 (19)
C4	0.061 (3)	0.042 (2)	0.074 (3)	−0.0055 (18)	0.025 (2)	−0.017 (2)
C5	0.063 (3)	0.066 (3)	0.064 (3)	−0.011 (2)	0.000 (2)	−0.019 (2)
C6	0.058 (2)	0.054 (2)	0.047 (2)	−0.0079 (19)	−0.0055 (18)	−0.0016 (19)
C7	0.053 (2)	0.0352 (18)	0.0312 (18)	−0.0039 (14)	0.0021 (16)	−0.0068 (13)
C8	0.060 (2)	0.041 (2)	0.0369 (19)	0.0011 (16)	0.0080 (17)	−0.0016 (15)
C9	0.068 (3)	0.0382 (19)	0.039 (2)	0.0048 (17)	−0.0056 (18)	−0.0041 (16)
C10	0.060 (3)	0.041 (2)	0.057 (2)	−0.0015 (17)	−0.0011 (19)	−0.0083 (18)
C11	0.061 (3)	0.057 (3)	0.056 (3)	0.0009 (19)	0.012 (2)	−0.0007 (19)
C12	0.062 (2)	0.049 (2)	0.036 (2)	−0.0008 (18)	0.0076 (18)	0.0022 (17)
C13	0.084 (3)	0.059 (3)	0.053 (3)	0.005 (2)	−0.011 (2)	0.015 (2)
C14	0.089 (4)	0.063 (3)	0.138 (6)	−0.023 (3)	0.041 (4)	−0.026 (4)
C15	0.100 (4)	0.057 (3)	0.050 (3)	0.016 (3)	−0.009 (3)	0.009 (2)
C16	0.069 (3)	0.065 (3)	0.098 (4)	0.003 (2)	−0.012 (3)	−0.006 (3)

### Geometric parameters (Å, °)

**Table:**

S1—O2	1.426 (3)	C8—H8	0.9300
S1—O1	1.427 (3)	C9—C10	1.401 (7)
S1—N1	1.636 (3)	C9—C15	1.511 (6)
S1—C1	1.775 (4)	C10—C11	1.391 (7)
N1—C7	1.441 (5)	C10—C16	1.487 (7)
N1—H1N	0.8600	C11—C12	1.377 (6)
C1—C6	1.374 (5)	C11—H11	0.9300
C1—C2	1.397 (6)	C12—H12	0.9300
C2—C3	1.389 (6)	C13—H13A	0.9600
C2—C13	1.501 (6)	C13—H13B	0.9600
C3—C4	1.378 (7)	C13—H13C	0.9600
C3—H3	0.9300	C14—H14A	0.9600
C4—C5	1.357 (8)	C14—H14B	0.9600
C4—C14	1.507 (7)	C14—H14C	0.9600
C5—C6	1.385 (7)	C15—H15A	0.9600
C5—H5	0.9300	C15—H15B	0.9600
C6—H6	0.9300	C15—H15C	0.9600
C7—C12	1.377 (6)	C16—H16A	0.9600
C7—C8	1.382 (5)	C16—H16B	0.9600
C8—C9	1.387 (6)	C16—H16C	0.9600
			
O2—S1—O1	119.5 (2)	C10—C9—C15	119.6 (4)
O2—S1—N1	106.57 (18)	C11—C10—C9	118.2 (4)
O1—S1—N1	105.59 (18)	C11—C10—C16	120.3 (5)
O2—S1—C1	106.50 (19)	C9—C10—C16	121.5 (5)
O1—S1—C1	111.18 (18)	C12—C11—C10	122.0 (5)
N1—S1—C1	106.75 (17)	C12—C11—H11	119.0
C7—N1—S1	120.4 (2)	C10—C11—H11	119.0
C7—N1—H1N	119.8	C11—C12—C7	119.3 (4)
S1—N1—H1N	119.8	C11—C12—H12	120.4
C6—C1—C2	120.6 (4)	C7—C12—H12	120.4
C6—C1—S1	116.5 (3)	C2—C13—H13A	109.5
C2—C1—S1	122.9 (3)	C2—C13—H13B	109.5
C3—C2—C1	116.6 (4)	H13A—C13—H13B	109.5
C3—C2—C13	118.6 (4)	C2—C13—H13C	109.5
C1—C2—C13	124.7 (4)	H13A—C13—H13C	109.5
C4—C3—C2	123.4 (4)	H13B—C13—H13C	109.5
C4—C3—H3	118.3	C4—C14—H14A	109.5
C2—C3—H3	118.3	C4—C14—H14B	109.5
C5—C4—C3	118.0 (4)	H14A—C14—H14B	109.5
C5—C4—C14	121.1 (5)	C4—C14—H14C	109.5
C3—C4—C14	120.9 (5)	H14A—C14—H14C	109.5
C4—C5—C6	121.2 (5)	H14B—C14—H14C	109.5
C4—C5—H5	119.4	C9—C15—H15A	109.5
C6—C5—H5	119.4	C9—C15—H15B	109.5
C1—C6—C5	120.1 (4)	H15A—C15—H15B	109.5
C1—C6—H6	120.0	C9—C15—H15C	109.5
C5—C6—H6	120.0	H15A—C15—H15C	109.5
C12—C7—C8	120.0 (4)	H15B—C15—H15C	109.5
C12—C7—N1	119.8 (4)	C10—C16—H16A	109.5
C8—C7—N1	120.1 (4)	C10—C16—H16B	109.5
C7—C8—C9	120.9 (4)	H16A—C16—H16B	109.5
C7—C8—H8	119.6	C10—C16—H16C	109.5
C9—C8—H8	119.6	H16A—C16—H16C	109.5
C8—C9—C10	119.6 (4)	H16B—C16—H16C	109.5
C8—C9—C15	120.8 (4)		
			
O2—S1—N1—C7	−56.1 (3)	C2—C1—C6—C5	−2.3 (7)
O1—S1—N1—C7	175.8 (3)	S1—C1—C6—C5	176.5 (4)
C1—S1—N1—C7	57.4 (3)	C4—C5—C6—C1	1.3 (8)
O2—S1—C1—C6	21.0 (4)	S1—N1—C7—C12	−104.3 (4)
O1—S1—C1—C6	152.8 (3)	S1—N1—C7—C8	76.6 (4)
N1—S1—C1—C6	−92.5 (3)	C12—C7—C8—C9	2.1 (6)
O2—S1—C1—C2	−160.2 (3)	N1—C7—C8—C9	−178.8 (3)
O1—S1—C1—C2	−28.4 (4)	C7—C8—C9—C10	−2.5 (6)
N1—S1—C1—C2	86.2 (3)	C7—C8—C9—C15	177.8 (4)
C6—C1—C2—C3	1.6 (6)	C8—C9—C10—C11	1.0 (6)
S1—C1—C2—C3	−177.2 (3)	C15—C9—C10—C11	−179.4 (4)
C6—C1—C2—C13	−178.8 (5)	C8—C9—C10—C16	−179.9 (4)
S1—C1—C2—C13	2.5 (6)	C15—C9—C10—C16	−0.2 (7)
C1—C2—C3—C4	0.2 (6)	C9—C10—C11—C12	1.1 (7)
C13—C2—C3—C4	−179.5 (5)	C16—C10—C11—C12	−178.1 (5)
C2—C3—C4—C5	−1.2 (7)	C10—C11—C12—C7	−1.6 (7)
C2—C3—C4—C14	179.2 (4)	C8—C7—C12—C11	0.0 (6)
C3—C4—C5—C6	0.5 (8)	N1—C7—C12—C11	−179.2 (4)
C14—C4—C5—C6	−180.0 (5)		

### Hydrogen-bond geometry (Å, °)

**Table:**

*D*—H···*A*	*D*—H	H···*A*	*D*···*A*	*D*—H···*A*
N1—H1N···O2^i^	0.86	2.41	2.976 (4)	124

Symmetry codes: (i) *x*, −*y*+1/2, *z*−1/2.
